# A Case Report of Intratesticular Hematoma in a Patient with Reiter’s Syndrome

**DOI:** 10.3390/diagnostics13121993

**Published:** 2023-06-07

**Authors:** Jia-Jyun Jhang, Szu-Ju Chen, Chi-Ping Huang, Huey-Yi Chen, Wei-Ching Lin, Yung-Hsiang Chen, Wen-Chi Chen

**Affiliations:** 1 Department of Urology, China Medical University Hospital, Taichung 404327, Taiwan; 2 Division of Urology, Department of Surgery, Taichung Veterans General Hospital, Taichung 407219, Taiwan; 3 School of Medicine, College of Medicine, China Medical University, Taichung 404333, Taiwan; 4 Department of Obstetrics and Gynecology, China Medical University Hospital, Taichung 404327, Taiwan; 5 Graduate Institute of Integrated Medicine, College of Chinese Medicine, China Medical University, Taichung 404333, Taiwan; 6 Department of Medical Imaging, China Medical University Hospital, Taichung 404327, Taiwan; 7 Department of Psychology, College of Medical and Health Science, Asia University, Taichung 413305, Taiwan; 8 Department of Medical Research, China Medical University Hospital, Taichung 404327, Taiwan

**Keywords:** Reiter’s syndrome, sulfasalazine, testicular hematoma, adverse drug reactions, inflammation

## Abstract

We present a case of a 28-year-old male patient with a spontaneous intratesticular hematoma. He had no history of trauma but experienced sudden onset of painful swelling in his right testis. Initially, testicular malignancy was suspected. The tumor marker of testis, including alfa-fetoprotein, lactic dehydrogenase, and β-human chorionic gonadotropin, was within normal range. The patient had been diagnosed with Reiter’s syndrome at the age of 20 and had been treated with sulfasalazine, non-steroidal anti-inflammatory drugs, and acetaminophen for eight years. Various imaging techniques before operation planning, including ultrasonography and computed tomography, revealed a hematoma that accounted for 32% of the testicular volume. During the waiting period before the operation, the patient was diagnosed with a hematoma and avoided a possible diagnosis of malignancy. Follow-up imaging with computed tomography and magnetic resonance imaging confirmed the presence of an intratesticular hematoma that had decreased in size. Since no other related factor contributed to this hematoma, and considering the possible hematological side effects of sulfasalazine, we suggest that this may be a rare side effect of sulfasalazine. Although the patient’s testis was preserved, further fertility should be observed because animal studies have reported that testicular hematoma may cause fertility changes if the initial volume occupied is over 30% of the testis.

## 1. Introduction

Reiter’s syndrome is a reactive arthritis with characteristics of conjunctivitis, urethritis, and arthritis [1,2,3]. Reactive arthritis is defined as an inflammatory synovitis presumed to be an infection-induced systemic illness in which no viable microorganisms can be found [4]. The immunopathogenesis of this disease is 80% linked to HLA-B27 and was reported to be associated with a human immuno-deficiency virus (HIV) [5,6]. Reiter’s syndrome is more severe and refractory to treatment in patients with HIV [5]. Symptoms of Reiter’s syndrome include joint pain, low back pain, urinary problems, eye inflammation, tendon inflammation, swelling of toes or fingers, and skin problems [7]. The cause remains unknown and the treatment is empiric [4]. Treatment of Reiter’s syndrome includes antibiotics, non-steroidal anti-inflammatory drugs (NSAIDs), corticosteroids, and immunosuppressive medicines such as methotrexate and sulfasalazine, etc. [8].

Intratesticular hematoma without rupture of the testis is rarely reported in the literature and is mainly caused by blunt trauma [9]. Acute hemorrhage of the testis may also be caused by direct infection with the SARS-CoV-2 virus, as shown in a recent study of hamsters [10]. However, spontaneous intratesticular hemorrhage is extremely rare without any identifiable risk factors [11].

We report a patient with spontaneous intratesticular hematoma without a history of trauma which has been treated with sulfasalazine for several months for Reiter’s syndrome.

## 2. Case Presentation

This is a case report of a 28-year-old male medical staff with a history of chronic reactive arthritis who presented with sudden onset of right testicular pain in January 2023. The patient was diagnosed with reactive arthritis at around 20 years old. His initial presentation was recurrent bilateral lower limb pain and swelling when he was about 17 years old. His symptoms, especially involving his plantar area and Achilles tendon, prompted him to initially seek medical aid from a physiatrist and orthopedist for pain control and rehabilitation programs. His serological profile, including Rheumatoid factor, ACPA, and ANA, were all within normal limits, making it difficult to diagnose his chronic arthritis. No specific pathogen infection was proved then. Persistent elevation of the Erythrocyte Sedimentation Rate (ESR) and C-Reactive protein (CRP) was noted despite medical treatment, and his symptoms persisted with fluctuation. After ruling out other arthritis differentials such as septic arthritis, rheumatoid arthritis, psoriatic arthritis, and inflammatory bowel disease-related arthritis, a rheumatologist eventually diagnosed him with reactive arthritis when he was approximately 20 years old. Since then, he has been in a relatively stable condition, experiencing limited episodes of arthritis attacks while undergoing treatment with Sulfasalazine. Over the past few years, several reevaluations for possible pathogens were conducted when his symptoms recurred, primarily manifesting as exacerbated pain in the plantar area and Achilles tendon. However, these studies yielded inconclusive results, which is a common scenario in chronic reactive arthritis patients. He is currently taking medication with Sulfasalazine 500 mg bis in die (twice a day (BID)), Ultracet (a compound of tramadol with acetaminophen), and acemetacin (an NSAID). He states that the arthritis tends to flare up, especially when he is under greater stress or fatigue, and he will add another 250 mg of Sulfasalazine if his symptoms worsen. His primary care rheumatologist has regularly monitored his ESR and CRP as reference indices for treatment. Nevertheless, adjustments to the medication dosage were primarily based on his symptoms rather than his laboratory data. According to the patient, there has been no involvement in the genital area, such as circinate balanitis. He also experienced erythema nodosum a few times, mainly involving his lower limbs. The erythema nodosum is self-limited and mostly fades out within weeks after presentation.

**Figure 1:**
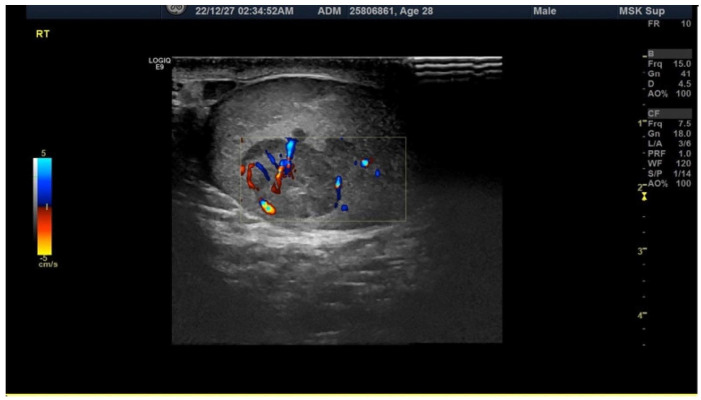
The figure shows an ecogram of the scrotum taken at the emergency department, revealing an irregular mass shadow on the right testis with a hypoechogenic density around the mass.

He had a sudden onset of right testis cramping pain when voiding. He had little sleep on his night shift the day before this pain attack. He noted that his right scrotum was larger than his left. The right testis pain did not accompany a fever, hematuria, nausea, or vomiting symptoms. He could void smoothly despite the pain, yet the pain persisted for hours, so he sought medical aid. There was no recent trauma history or sexual activity. Physical examination showed an irregular surface intratesticular mass around 1.5 cm in his right scrotum with tenderness. No change in his scrotum skin color was seen. His prepuce and glans were in normal appearance without tenderness, ulceration, or redness. The pain soon subsided after his hospital visit. Scrotal sonography demonstrated a mixed-echoic mass in his right testis, measured at 1.3 cm and accounted for 32% of the testis volume, with the preserved flow of bilateral testis (Figure 1).

**Figure 2:**
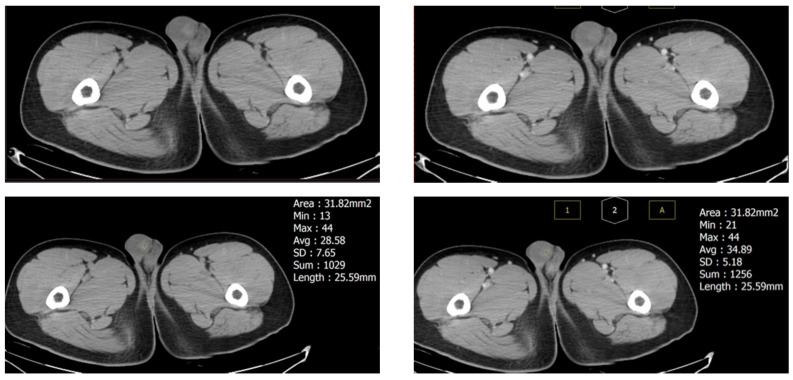
Computed tomography of the testis nine days after the first attack revealed a 13 mm hyperdense mass (32% of testicular volume) without enhancement, which suggests a hematoma. The size of the testis had decreased compared to the first attack.

Urine analysis showed no specific findings, and there was no hematuria or pyuria. Laboratory examinations, including Blood Cell Count, activated partial thromboplastin time, Prothrombin Time, α-fetoprotein, β-human chorionic gonadotropin (HCG), and lactate dehydrogenase (LDH) were all within the normal limit. For further evaluation, contrast computed tomography (CT) was performed nine days after the first attack, showing a 13 mm high-density nodule in the right testis without enhancement. No enlarged lymph nodes were found in his abdomen or pelvis (Figure 2).

**Figure 3:**
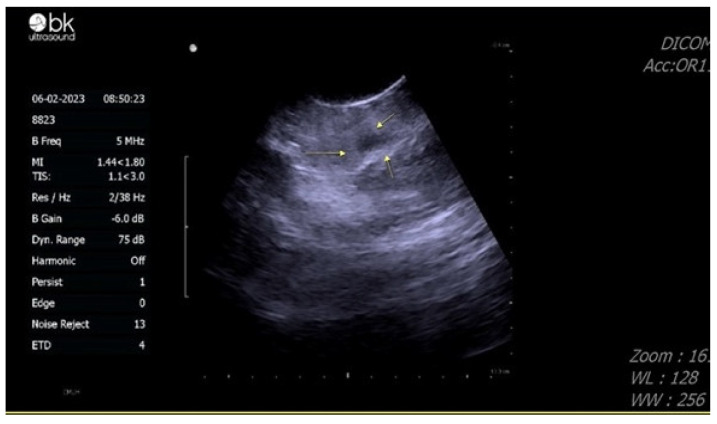
Echogram of the testis performed one month after the emergency room visit, showing the mass displayed a decrease in size and became more hypoechoic compared to previous images.

A malignant testis tumor was suspected due to scrotal echo findings, and radical orchidectomy was suggested. The patient hesitated about the operation, and when he finally decided to receive the operation, it was about six weeks after the discovery of the testis mass. He preserved his sperm first before the operation. Sperm analysis showed that his sperm count was 92 × 10^6^/mL, total motility in the first hour was 60%, progressive motility was 39%, and normal form was 1%. Scrotal sonography was performed again before the operation in February 2023 (Figure 3).

**Figure 4:**
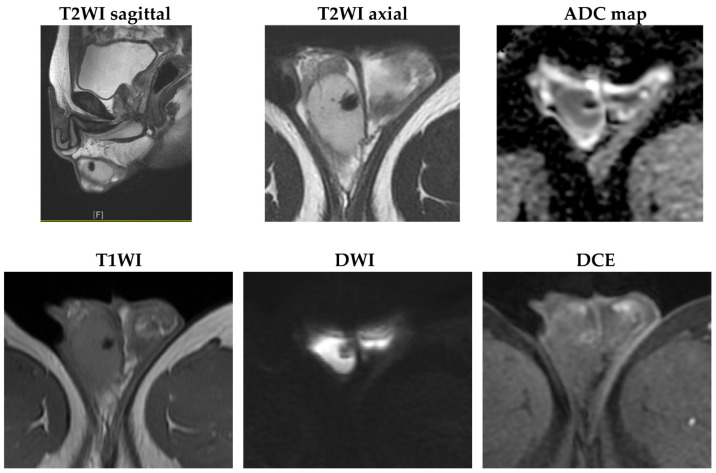
MRI images taken three months after the first attack revealed that the mass size had decreased to 7 mm. Hemosiderin deposits showed low signal intensity on all sequence images, with a rim-like reactive enhancement and reactive hydrocele (T2WI: T2-weighted image; ADC: apparent diffusion coefficient; T1WI: T1-weighted image; DWI: diffusion-weighted imaging; DCE: dynamic contrast-enhanced).

The mass showed a decrease in size and became more hypoechoic compared with previous images. Based on his CT image and the change in scrotal sonography, a testicular hematoma was suspected. Magnetic resonance imaging (MRI) was arranged 12 weeks after the pain attack, showing a decrease in mass size to 7 mm, with deposition of hemosiderin and reactive hydrocele, providing further evidence that the testis mass is a testicular hematoma (Figure 4).

As this is a suspected ADR, we utilized the Naranjo drug causality algorithm to evaluate the suspected drug in the case results. The Naranjo algorithm generates a score ranging from negative four to thirteen, where a score of zero suggests a doubtful ADR, and a score of nine or higher indicates a definite ADR [12]. Our evaluation yielded a score of five, indicating a probable ADR. While the Naranjo probability scale does not exclude severe ADRs from the assessment, these cases may receive a lower achievable score individually due to certain items being unable to be scored positively. For example, re-challenging for severe ADRs may often be unsafe and, therefore, not feasible. Thus, the assessment may only result in a possible or probable causality score and should never be considered definitive (final score ≥ 9). In our case, caution is necessary when interpreting the final score, and dismissing it as only a probable ADR without considering its severity should be avoided [13]. In addition, the case has been notified to a pharmacovigilance system (Taiwan National Adverse Drug Reactions Reporting System); the notification number (Worldwide ID) of the ADR mentioned in the article is “TW-TFDA-TDS-1120003247”. After a discussion with a rheumatologist, the dosage of sulfasalazine has been decreased. The patient is under monitoring for any possible adverse event.

## 3. Discussion

Due to a delayed operation schedule and repeated imaging with CT and ultrasonography, the patient was able to preserve his testis. There was no history of trauma or evidence of a bleeding tendency from laboratory results. Tracing back the patient’s history, he had only been taking long-term medication with sulfasalazine, NSAIDs, and Ultracet. Since sulfasalazine may have the side effect of causing hemolytic disorders [14], we infer that this spontaneous intratesticular hematoma could be a side effect of sulfasalazine. This is the first reported case of this unusual side effect of sulfasalazine in a patient with Reiter’s syndrome.

Reiter’s syndrome may affect fertility [15]. In a study of 628 questionnaires regarding childbearing in Reiter’s syndrome, it was reported that men diagnosed before the age of 30 had fewer children on average than those diagnosed after the age of 30. The report also found that men below the age of 40 had more medical evaluations for fertility than those over 40. The study indicated that being diagnosed with Reiter’s syndrome during one’s fertile years may impact fertility. In addition to the disease itself, sulfasalazine may have a side effect of infertility [16]. Cosentino et al. compared ten patients who had been treated with sulfasalazine for chronic inflammatory bowel disease for over five years with 19 normal controls and found poor sperm quality and sex-related hormone levels. Fertility may reverse after stopping the drug. Although our patient has been treated with sulfasalazine for over five years and still has normal semen analysis, future fertility status remains to be followed for both drug and disease complications.

Focusing on the cause of the spontaneous testicular hematoma, the patient denied any recent trauma, sexual activity, or other possible medical interventions that could cause a hematoma. CT scans did not reveal any other possible secondary causes of his hematoma [17,18]. Reviewing the patient’s clinical course, we paid close attention to his underlying reactive arthritis and medication use. Sulfasalazine is a disease-modifying anti-rheumatic drug (DMARD) that is widely used in inflammatory bowel disease (IBD), rheumatoid arthritis (RA), ulcerative colitis, Crohn’s disease, polyarticular juvenile idiopathic arthritis, reactive arthritis, ankylosing spondylitis, psoriasis, and several autoimmune-related diseases [19,20]. A Cochrane database systematic review by Suarez-Almazor et al. estimated that short-term treatment with sulfasalazine reduced pain scores for rheumatoid arthritis by nearly half [21]. The withdrawal rate of sulfasalazine due to lack of efficacy was low. However, patients may drop out due to side effects [22,23]. The major side effects were gastrointestinal symptoms (10%), mucocutaneous reactions (7%), and hematological abnormalities (2%). Despite its treatment effect on these diseases, the drug is also known to have several side effects, including hemolytic anemia, agranulocytosis, male infertility, and hepatotoxicity [24,25,26,27]. Side effects of sulfasalazine involve many organs. Regarding the hematological side effects of sulfasalazine, there have been reports of leukemia, hemolytic anemia, and low platelet count. Therefore, we have a high suspicion that the patient’s intratesticular hematoma may be related to his use of sulfasalazine. Further studies are still needed to better understand the possible relationship between testicular hematoma and sulfasalazine use and its possible mechanism.

Intratesticular hematoma is most commonly found in the setting of scrotal trauma. Iatrogenic causes, such as after a testis biopsy or vasectomy, have also been reported. Although rare, there have been some reports describing idiopathic spontaneous intratesticular hematoma. In these cases, a specific cause of the intratesticular hematoma could not be found, and malignancy was often the clinician’s first impression and concern. Using sonography images, it is difficult to differentiate an intratesticular hematoma from a malignancy. Hence, the diagnosis is often made after orchiectomy [11,28,29].

This patient highlights the importance of including spontaneous testicular hematoma in our differential diagnosis. As previously mentioned, intratesticular hematomas are often diagnosed after orchiectomy since their imaging findings may mimic those of malignant testicular tumors. Furthermore, apart from traumatic or iatrogenic causes, drug-related side effects may also be a possible cause of testicular hematoma, and further study is needed to investigate its possible mechanism.

For imaging studies of non-malignant lesions of the testis, Bhatt et al. suggested that grayscale and color-flow Doppler ultrasonography help visualize the characteristics of benign lesions [30,31]. Bhatt et al. also proposed that MRI is a helpful imaging tool. Doppler ultrasonography and careful history-taking are ideal tools for diagnosing intratesticular hematoma [32,33], which can reduce the chance of orchiectomy.

As for our patient, malignancy was still our first impression, and we scheduled an orchiectomy for him. Fortunately, sonography performed before the operation demonstrated that the mass had decreased in size and became more hypoechoic. These changes in the sonographic image findings indicated that the mass might be an intratesticular hematoma instead of a malignant tumor, preventing the patient from undergoing an orchiectomy. Follow-up MRI confirmed the diagnosis, showing a gradual decrease in the size of the hematoma. However, long-term results should be further observed, especially regarding infertility. The patient has preserved his sperm, and we can also recheck his semen analysis before he gets married to make further comparisons. This may help determine whether the decision to perform an orchiectomy is adequate.

A hematoma of the testis has the potential to cause infertility. Aminsharifi et al. conducted a rat animal study on the intratesticular hematoma [34]. They studied the effects of blunt trauma on the testis in male rats. The rats’ testes were injected with different volumes of autologous blood to evaluate the effects of volume. The results indicated that injecting equivalent testicular volumes of 30% and 40% autologous blood could cause a reduction in spermatogenesis, Sertoli cells, Leydig cells, and blood testosterone levels after 60 days. Therefore, a hematoma occupying over 30% of the testicular volume may have a detrimental effect on fertility. Our patient presented with a hematoma occupying 32% of his testicular volume. This may highlight the need to further observe this patient’s future fertility potential.

One of the drugs used to treat Reiter’s syndrome is NSAIDs. There are reports that NSAIDs may block cyclooxygenase (COX) and cause platelet dysfunction [35]. Gastrointestinal (GI) hemorrhage is a frequently reported side effect of NSAIDs [36,37]. However, hemorrhage outside the GI tract is extremely rare unless the patient also takes antiplatelet drugs [38]. Our patient had been taking NSAIDs and sulfasalazine, but his platelet function was normal. He did not have any GI tract hemorrhage. Although NSAIDs may cause hemorrhage, there have been no reports of spontaneous intratesticular hematoma in the literature. Since the chance of generalized hematological side effects from sulfasalazine is higher than from NSAIDs, sulfasalazine is the first consideration as the cause of the intratesticular hematoma because of its potentially serious hematological side effects [39].

In conclusion, we report a case of spontaneous intratesticular hematoma that may have been caused by a rare side effect of sulfasalazine. There was no other possible cause of the hematoma. Spermatogenesis at the time of presentation was normal. However, further observation of the patient’s fertility status is warranted to assess the long-term effects of the drug or disease itself and to determine whether to perform an orchiectomy on a hematoma occupying over 30% of the testicular volume.

## Data Availability

All data are available upon request to the corresponding author.
